# The Relationship of the Plasma Glycated CD59 Level with Microvascular Complications in Diabetic Patients and Its Evaluation as a Predictive Marker

**DOI:** 10.3390/jcm14134588

**Published:** 2025-06-28

**Authors:** Ozgur Yilmaz, Osman Erinc, Ayca Gul Gungordu, Mehmet Erdogan, Murvet Algemi, Murat Akarsu

**Affiliations:** 1 Department of Internal Medicine, Kanuni Sultan Suleyman Training and Research Hospital, 34303 Istanbul, Turkey; doctorerinc@gmail.com (O.E.); muratakarsu79@gmail.com (M.A.); 2 Department of Neurology, Kanuni Sultan Suleyman Training and Research Hospital, 34303 Istanbul, Turkey; aycagul@yahoo.com; 3 Department of Ophthalmology, Kanuni Sultan Suleyman Training and Research Hospital, 34303 Istanbul, Turkey; drgozmerdogan@gmail.com; 4 Department of Clinical Biochemistry, Kanuni Sultan Suleyman Training and Research Hospital, 34303 Istanbul, Turkey; algemimurvet@gmail.com

**Keywords:** type 2 diabetes mellitus, glycated CD59, microvascular complications, diabetic retinopathy, diabetic nephropathy, diabetic neuropathy

## Abstract

**Background/Objectives:** Type 2 diabetes mellitus (T2DM) is a prevalent metabolic disease characterized by chronic hyperglycemia and progressive microvascular complications, including retinopathy, nephropathy, and neuropathy. While traditional markers like HbA1c capture average glycemic control, they often fail to predict microvascular damage risk. Glycated CD59 (GCD59), a complement regulatory protein modified under hyperglycemic conditions, has emerged as a promising biomarker reflecting complement dysregulation and endothelial injury. This study aimed to examine the relationship between plasma GCD59 levels and the presence of microvascular complications in patients with type 2 diabetes mellitus and to evaluate whether GCD59 shows potential for future use as a predictive biomarker, pending prospective validation. **Methods:** In this single-center, prospective case–control study, 246 participants were enrolled: 82 healthy controls, 82 T2DM patients without microvascular complications (DM − MC), and 82 T2DM patients with microvascular complications (DM + MC). Microvascular complications were defined based on standardized criteria for retinopathy, nephropathy, and neuropathy. Plasma GCD59 levels were measured using validated ELISA methods. Receiver operating characteristic (ROC) analyses, forest plots, and odds ratio calculations were employed to assess the discriminatory performance of GCD59. Statistical significance was set at *p* < 0.05. **Results:** Plasma GCD59 levels were significantly elevated across all diabetic groups compared to healthy controls (*p* < 0.001), with the highest levels in the DM + MC group (median 4.5 ng/mL) versus DM − MC (median 1.9 ng/mL) and controls (median 1.2 ng/mL). ROC analysis demonstrated excellent diagnostic performance for distinguishing DM + MC from healthy controls (AUC = 0.946, sensitivity 89%, specificity 97.6%) and good performance for distinguishing DM + MC from DM − MC (AUC = 0.849, sensitivity 72%, specificity 87.8%). Forest plot analyses confirmed significantly elevated odds ratios for GCD59 across all microvascular subgroups. Importantly, GCD59 levels correlated positively with inflammatory markers (CRP, ESR, leukocyte count), suggesting a combined role of complement dysregulation and chronic inflammation in diabetic microangiopathy. **Conclusions:** Plasma GCD59 may be a promising biomarker for identifying T2DM patients who may be at increased risk for microvascular complications, independent of conventional glycemic markers. Given the cross-sectional design of this study, causal inference is not possible; prospective validation is required. The observed strong discriminatory performance highlights potential future clinical utility, pending further validation of diagnostic thresholds, assay standardization, and feasibility in routine care settings.

## 1. Introduction

Type 2 diabetes mellitus (T2DM) is a globally prevalent metabolic disorder characterized by impaired pancreatic β-cell insulin secretion and peripheral insulin resistance [1]. Beyond hyperglycemia, T2DM is linked to microvascular and macrovascular complications that significantly increase morbidity and mortality. Microvascular damage, such as retinopathy, nephropathy, and neuropathy, is driven by endothelial dysfunction, oxidative stress, and chronic low-grade inflammation. These multifactorial mechanisms disrupt vascular tone, promote capillary permeability and microthrombus formation, and ultimately lead to irreversible structural changes in target organs [2,3].

Although many molecular mechanisms underlying diabetic vascular damage have been elucidated, the complement system has recently gained attention as a critical yet underexplored contributor. As a central component of the innate immune response, the complement cascade mediates inflammation, cell lysis, and tissue remodeling. Increasing evidence from clinical and experimental studies suggests that dysregulation of complement regulatory proteins plays a significant role in the development of diabetes-related complications [4]. Studies conducted in both human and animal models have demonstrated that complement proteins contribute to vascular occlusion and the development of both microvascular and macrovascular complications in diabetes by promoting a chronic inflammatory state and a pro-thrombotic environment [5].

Hyperglycemia has been shown to alter the balance of complement activation, reducing levels of C1q and C3 while increasing C3b concentrations. This pattern suggests that elevated glucose may preferentially activate the classical complement pathway while suppressing the alternative pathway, further supporting a mechanistic link between hyperglycemia and complement dysregulation [6].

CD59 is a membrane-bound glycoprotein anchored by a glycosylphosphatidylinositol linkage, essential for regulating the terminal complement pathway and preventing complement-mediated lysis of host cells. By binding complement components C8 and C9, CD59 blocks membrane attack complex (MAC) formation and C9 polymerization. A soluble, cleaved form of CD59 has also been identified in plasma and urine, reflecting its release from cell membranes [7,8]. Under chronic hyperglycemia, CD59 undergoes non-enzymatic glycation, primarily at lysine 41, producing glycated CD59 (GCD59), a structurally modified and functionally inactive form [9,10]. This inactivation promotes MAC accumulation, inducing cytotoxicity, inflammation, thrombosis, and aberrant cell proliferation, particularly in diabetic tissues such as the kidney, nerves, and vasculature, where GCD59 and MAC colocalize. Soluble GCD59, released into circulation, may serve as a biomarker reflecting cumulative glycation burden [11,12].

Although CD59’s role in inhibiting MAC formation is well established, evidence supporting its broader use as a general biomarker of inflammation or disease remains limited [4]. Recent studies suggest that plasma GCD59 levels may serve not only as a diagnostic marker for diabetes but also as a prognostic indicator, highlighting its potential as both a biomarker and therapeutic target in diabetic vascular pathology [10,13]. Although CD59 glycation and complement activation are increasingly linked to diabetic vascular complications, the clinical utility of GCD59 as a biomarker remains unclear. Prior studies have mostly addressed its diagnostic potential, with limited data on its association with specific microvascular complications or its prognostic value in patients with established disease. While glycated Hb (HbA1c) is the current gold standard for assessing chronic glycemia, it primarily reflects average glucose over the past 8–12 weeks and may be influenced by conditions affecting erythrocyte turnover, such as anemia, hemoglobinopathies, or chronic kidney disease [14,15,16]. Fructosamine reflects shorter-term glycemia (2–3 weeks), but is confounded by variations in serum protein concentration and lacks predictive utility for complications [17]. Advanced glycation end-products are chemically stable indicators of cumulative glycemic stress and oxidative damage, yet their clinical application remains limited by methodological variability and lack of standardized assays [18]. Urine albumin-to-creatinine ratio (UACR) is widely used to detect incipient diabetic nephropathy; however, it is susceptible to acute changes in hydration, exercise, and urinary tract conditions and provides little information about non-renal microvascular injury [19]. Unlike these markers, GCD59 is not only a product of hyperglycemia but also functionally involved in the pathogenesis of diabetic vascular injury through complement activation and MAC formation [11,12]. Accordingly, GCD59 may serve as an integrated marker that reflects both metabolic dysregulation and endothelial dysfunction in the context of diabetic vascular complications.

To the best of our knowledge, this is the first study in the literature to directly evaluate the relationship between plasma GCD59 levels and microvascular complications in patients with diabetes. By addressing this critical gap, the present study aims to expand our understanding of GCD59 within the context of diabetic vasculopathy and to assess its potential as a novel biomarker and therapeutic target in diabetes care.

## 2. Materials and Methods

### 2.1. Study Design and Setting

This prospective study included 246 participants, comprising 164 adult patients diagnosed with T2DM, aged between 18 and 80 years, who presented to the internal medicine outpatient clinics of our hospital, and 82 healthy individuals without any known disease or complaints, all of whom were prospectively recruited between 1 January 2025 and 1 May 2025, from the outpatient internal medicine clinic of our institution. Healthy control participants (*n* = 82) were selected from individuals attending routine check-ups at our outpatient clinic. They were matched at the group level for age and sex with diabetic patients. Controls had no history of diabetes mellitus, cardiovascular disease, microvascular or macrovascular complications, or chronic inflammatory or autoimmune conditions. Individuals receiving glucose-lowering, lipid-lowering, antihypertensive, corticosteroid, or immunosuppressive medications were excluded. Body mass index (BMI) values were recorded and considered as a potential confounder during statistical analyses. The study population was divided equally into three groups (*n* = 82 in each group) to ensure balanced statistical comparisons and minimize group size-related bias: healthy controls (Group 1), patients with T2DM without microvascular complications (Group 2), and patients with T2DM with microvascular complications (Group 3). Microvascular complications were defined as the presence of retinopathy, nephropathy, or neuropathy.

The diagnosis of T2DM was established according to the American Diabetes Association 2024 guidelines, requiring at least one of the following criteria: fasting plasma glucose ≥ 126 mg/dL, 2-h plasma glucose ≥ 200 mg/dL during an oral glucose tolerance test, or HbA1c ≥ 6.5% [20].

Diabetic retinopathy was assessed by an experienced ophthalmologist following a standardized protocol. After pupillary dilation with 1% tropicamide, biomicroscopic indirect ophthalmoscopy was performed using a 90D Volk lens, and color fundus photographs were obtained. Retinopathy severity was graded based on the International Clinical Diabetic Retinopathy Severity Scale. Macular edema was evaluated using optical coherence tomography and classified according to the Early Treatment Diabetic Retinopathy Study criteria. Fundus fluorescein angiography was performed in patients with suspected proliferative retinopathy. Diagnoses and classifications were conducted according to the American Academy of Ophthalmology 2023 guidelines, with independent evaluations by two ophthalmologists in cases of diagnostic uncertainty [21].

Diabetic neuropathy was evaluated by an experienced neurologist. All patients underwent screening using the Michigan Neuropathy Screening Index (MNSI) and the Toronto Clinical Neuropathy Score (TCNS). Patients with an MNSI score ≥ 2 and a TCNS score ≥ 6 were selected for further assessment. Sensory neuropathy was additionally evaluated using the 10 g monofilament test and vibration testing on the feet. Electrophysiological assessments included electromyography and nerve conduction studies, measuring sensory conduction velocities in the median, ulnar, and sural nerves, and motor conduction in the tibial, peroneal, median, and ulnar nerves. Mixed neuropathy was diagnosed when both sensory and motor abnormalities were present. Polyneuropathy was confirmed by identifying significant reductions or delays in nerve conduction velocities. Final diagnoses were established by integrating clinical findings and electrophysiological results, following the American Academy of Neurology 2023 and Toronto Consensus Criteria [22].

Diabetic nephropathy was diagnosed in accordance with the Kidney Disease: Improving Global Outcomes 2023 criteria, defined by a urine albumin-to-creatinine ratio ≥ 30 mg/g on two measurements within three months and/or an estimated glomerular filtration rate (eGFR) < 60 mL/min/1.73 m^2^, calculated using the Chronic Kidney Disease Epidemiology Collaboration (CKD-EPI) formula [23]. Diabetic nephropathy was identified based on persistent urine albumin-to-creatinine ratio values between 30 and 300 mg/g (moderately increased albuminuria) or values exceeding 300 mg/g (severely increased albuminuria) on repeated measurements.

### 2.2. Exclusion Criteria

Patients were excluded if they met any of the following criteria: type 1 diabetes mellitus, diabetic ketoacidosis, severe renal, hepatic, cardiovascular, and neurological disease; acute or chronic infections or chronic pulmonary conditions. Other exclusion factors included thyroid disorders, steroid use, immune system disorders, a history of ocular or retinal surgery, malignancy, malnutrition, and pregnancy or breastfeeding. These exclusion criteria were applied uniformly to ensure a homogeneous study population and minimize potential confounding variables.

### 2.3. Data Collection

Demographic and clinical characteristics, including age, gender, family history of diabetes, and duration since diabetes mellitus diagnosis, were recorded. BMI was calculated as weight in kilograms divided by the square of height in meters, and patients were categorized based on standard BMI classifications.

Systolic and diastolic blood pressure measurements were obtained from the right arm using a mechanical sphygmomanometer after a 15-minute rest. Medication use was recorded, and the presence of retinopathy, nephropathy, neuropathy, cardiovascular disease, cerebrovascular disease, peripheral vascular disease, dyslipidemia, hypertension, and metabolic syndrome was documented.

### 2.4. Blood Sampling and Biochemical Analysis

Fasting venous blood samples were collected between 8:00 and 10:00 a.m. after an overnight fasting period of 10–12 h. Blood was drawn from the brachial vein in the antecubital fossa into both plain and anticoagulant-free tubes. Laboratory staff conducting the analyses were blinded to participant groupings. Samples were centrifuged at 4 °C for 10 min at 4000 rpm, and biochemical analyses were performed immediately thereafter. Fasting plasma glucose, creatinine, alanine aminotransferase, albumin, lipid profile (measured using enzymatic colorimetric methods), and C-reactive protein (CRP), determined via immunoturbidimetric assay) were analyzed using the Roche Cobas 8000 c 702 analyzer (Roche Diagnostics, Mannheim, Germany). HbA1c was measured by high-performance liquid chromatography using the ARKRAY/ADAMS HA-8180V system (ARKRAY Inc., Kyoto, Japan). Thyroid-stimulating hormone and insulin levels were assessed using electrochemiluminescence on the Roche Cobas E801 analyzer (Roche Diagnostics, Mannheim, Germany). Plasma aliquots designated for additional analyses were promptly frozen and stored at −80 °C until further evaluation.

### 2.5. Measurement of Plasma Glycated CD59

In addition to routine biochemical analyses, plasma GCD59 levels were measured as the primary biomarker of interest in this study. Venous blood samples were collected, centrifuged, and the resulting plasma was aliquoted into Eppendorf tubes and stored at −80 °C until analysis. Plasma GCD59 concentrations were determined using a commercially available human enzyme-linked immunosorbent assay (ELISA) kit (Human Plasma Glycated CD59, Byabscience Biological Technology Co., Nanjing, China) following the manufacturer’s protocol. Measurements were conducted on an ELX800DA microplate reader (Diagnostic Automation Inc., Woodland Hills, CA, USA), with data acquisition via the KC Junior software program (Agilent Technologies, Santa Clara, CA, USA), and all samples were analyzed in duplicate. The assay procedure included preparation of reagents, loading 50 μL of standards and plasma samples into the wells, followed by the addition of 100 μL of enzyme conjugate (excluding blank wells). Plates were incubated at 37 °C for 60 min, washed four times, and then treated with 50 μL each of Substrate A and Substrate B, followed by a 15 min incubation at 37 °C protected from light. The reaction was terminated by adding 50 μL of the stop solution, and the optical density was measured at 450 nm. Assay performance characteristics included an intra-assay coefficient of variation (CV) of <10%, an inter-assay CV of <15%, a sensitivity of <0.1 ng/mL, and a quantifiable range of 0.75 ng/mL to 24 ng/mL. No cross-reactivity was observed.

### 2.6. Outcomes

The primary outcome of this study was to evaluate the association between plasma GCD59 levels and the presence of microvascular complications, including diabetic retinopathy, nephropathy, and neuropathy, in patients with T2DM. Secondary outcomes included assessing the correlation between GCD59 levels and the severity of microvascular complications, evaluating the diagnostic performance of GCD59 through receiver operating characteristic (ROC) curve analysis with optimal cut-off determination, and analyzing the association between GCD59 levels and microvascular complications by calculating odds ratios, visualized using forest plots.

### 2.7. Statistical Analysis

Statistical analyses were performed using IBM SPSS Statistics version 28.0 (IBM Corporation, Armonk, NY, USA) and the R programming language (version 3.6.1). The distribution of continuous variables was assessed using the Shapiro–Wilk test. For variables with normal distribution, comparisons between groups were performed using Student’s t-test or one-way analysis of variance (ANOVA) with Tukey’s post hoc test. For variables not showing normal distribution, the Mann–Whitney U test or Kruskal–Wallis test with Dunn’s post hoc correction was applied. Categorical variables were analyzed using the chi-square test. Odds ratios were calculated for subgroup comparisons and visualized using forest plots. ROC curve analyses were conducted to evaluate diagnostic performance, with the optimal cut-off value determined using the Youden index. Continuous data were reported as mean ± standard deviation for normally distributed variables or median (interquartile range) for non-normally distributed variables. Categorical variables were presented as percentages. A *p*-value of <0.05 was considered statistically significant. Two multivariate logistic regression models were constructed to evaluate the independent association between plasma GCD59 and microvascular complications. Model 1 compared healthy controls with diabetic patients presenting microvascular complications (control vs. DM + MC) and adjusted for age, BMI, systolic and diastolic blood pressure, glucose, HbA1c, eGFR, low density lipoprotein cholesterol (LDL), erythrocyte sedimentation rate (ESR), C-reactive protein (CRP), leukocyte count, and UACR. Model 2 contrasted diabetic patients without and with microvascular complications (DM − MC vs. DM + MC), adjusting for diabetes duration, glucose, urea, eGFR, ESR, leukocyte count, and UACR. Variables for multivariable logistic regression were selected based on both clinical relevance and a univariate *p*-value < 0.25. Prior to model fitting, collinearity among candidate variables was assessed. A stepwise forward likelihood ratio method was applied to construct the final models, and adjusted odds ratios with 95% confidence intervals were reported, using a significance threshold of *p* < 0.05.

## 3. Results

A total of 246 participants were included in the study, consisting of 82 healthy controls, 82 patients with T2DM without microvascular complications (DM − MC), and 82 patients with microvascular complications (DM + MC). In the DM + MC group (*n* = 82), patients were categorized into four distinct subgroups: retinopathy (*n* = 25; 30.5%), neuropathy (*n* = 12; 14.6%), nephropathy (*n* = 12; 14.6%), and the mixed group (*n* = 33; 40.2%), defined as patients exhibiting two or more concurrent microvascular complications. Of the total cohort, 129 were female and 117 were male.

Age and gender distribution were similar across the groups (*p* = 0.098 and *p* = 0.254, respectively). BMI was significantly higher in both diabetic groups compared to controls (*p* < 0.001), while no significant difference was found between DM − MC and DM + MC. In addition, both systolic and diastolic blood pressure values were higher in diabetic groups compared to controls (*p* < 0.05), but not significantly different between DM − MC and DM + MC. Table 1 summarizes the demographic, anthropometric, and clinical data across the study groups.

As shown in Table 2, plasma GCD59 levels differed significantly among all groups (*p* < 0.001). Median GCD59 values were 1.2 ng/mL in healthy controls, 1.9 ng/mL in DM − MC, and 4.5 ng/mL in DM + MC. Post hoc analyses confirmed significantly higher GCD59 levels in DM + MC compared to both DM − MC (*p* < 0.001) and control groups (*p* < 0.001), and a significant difference between DM − MC and controls (*p* = 0.001). Significant differences in metabolic and other laboratory parameters were observed among the study groups (Table 2). Fasting glucose, HbA1c, and homeostasis model assessment of insulin resistance (HOMA-IR) levels were significantly elevated in both diabetic groups (DM − MC and DM + MC) compared to healthy controls (*p* < 0.001 for all), with no significant difference between the diabetic groups (*p* > 0.05). Serum creatinine was significantly higher, while eGFR was significantly lower in the DM + MC group compared to both other groups (*p* < 0.001). In addition, a statistically significant difference in urine albumin-to-creatinine ratio was observed among the three groups (*p* < 0.001).

Inflammatory parameters, including CRP, ESR, and leukocyte count, were also significantly elevated in the DM + MC group (*p* < 0.001 for all), with ESR also elevated in the DM − MC group relative to controls (*p* < 0.05). Moreover, LDL cholesterol levels showed a decreasing trend across the groups and differed significantly, with the highest levels in controls and the lowest in the DM + MC group (*p* = 0.001).

Among patients with microvascular complications (DM + MC), plasma GCD59 levels were significantly elevated across all complication subtypes, including retinopathy, neuropathy, nephropathy, and mixed forms, compared to both healthy controls and the DM − MC group (*p* < 0.05 for all; Table 3). Median GCD59 concentrations were 4.6 ng/mL in retinopathy, 4.3 ng/mL in neuropathy and nephropathy, and 4.5 ng/mL in the mixed complication group. Although these levels were markedly higher relative to non-complicated diabetic patients (median: 1.9 ng/mL) and healthy controls (median: 1.2 ng/mL), no statistically significant differences were observed among the four microvascular complication subgroups (*p* > 0.05). A comprehensive comparison of GCD59 distributions across all groups is summarized in Table 3.

**Figure 1:**
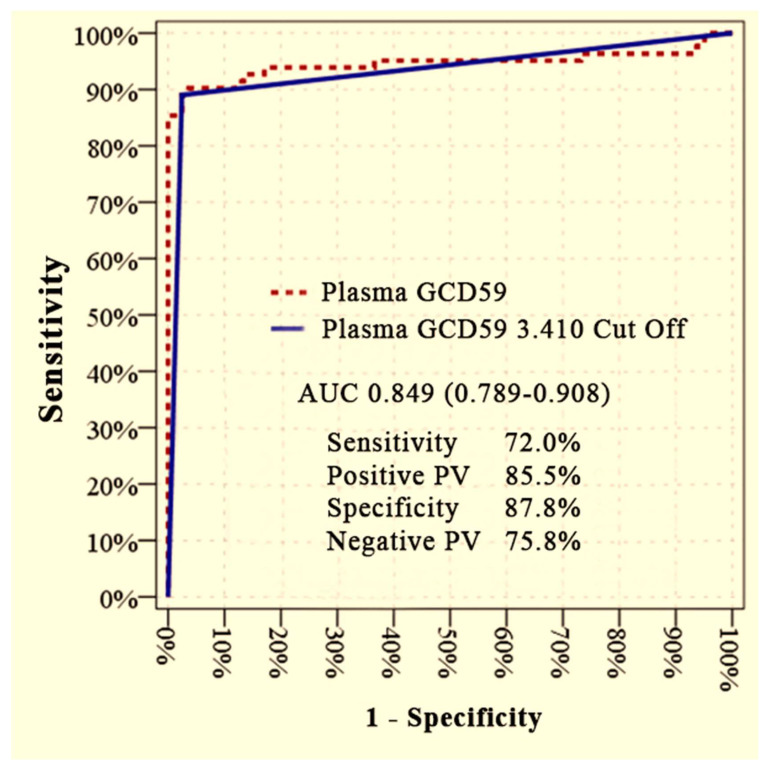
ROC curve analysis to predict the presence of microvascular complications in diabetic patients (DM + MC vs. DM − MC). AUC: area under curve, PV: predictive value.

Plasma GCD59 levels effectively differentiated diabetic patients with microvascular complications (DM + MC) from those without complications (DM − MC), as demonstrated by ROC curve analysis. The area under the curve (AUC) was 0.849 (95% CI: 0.789–0.908) at an optimal cut-off value of 3.410 ng/mL, yielding a sensitivity of 72.0%, specificity of 87.8%, positive predictive value (PPV) of 85.5%, and negative predictive value (NPV) of 75.8% (Figure 1).

**Figure 2:**
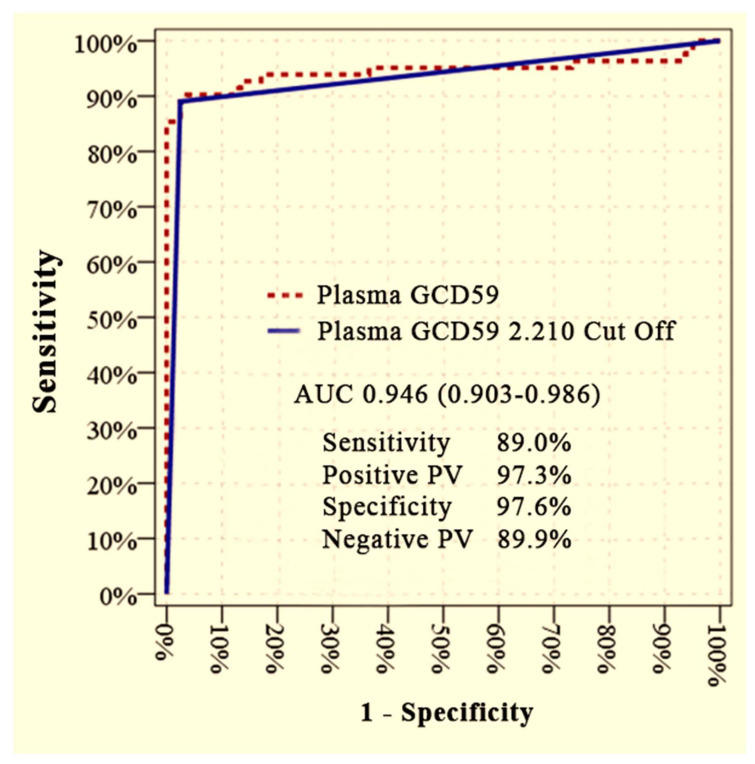
ROC curve analysis to distinguish diabetic patients with microvascular complications (DM + MC) from healthy controls. AUC: area under curve, PV: predictive value.

Furthermore, plasma GCD59 levels showed excellent discriminatory performance in distinguishing patients with microvascular complications from healthy controls. An AUC of 0.946 (95% CI: 0.903–0.986) was achieved at a cut-off value of 2.210 ng/mL, corresponding to a sensitivity of 89.0%, specificity of 97.6%, PPV of 97.3%, and NPV of 89.9% (Figure 2).

Multivariate logistic regression analysis was performed to assess the independent association between plasma GCD59 levels and the presence of microvascular complications. In Model 1 (Control vs. DM + MC), GCD59 was independently associated with microvascular complications after adjustment for age, BMI, systolic and diastolic blood pressure, glucose, HbA1c, eGFR, LDL, ESR, CRP, leukocyte count, and UACR (adjusted OR = 21.48; 95% CI: 3.505–131.62; *p* = 0.001). The results of Model 1 are presented in Table 4.

In Model 2 (DM − MC vs. DM + MC), GCD59 also remained significantly associated with microvascular complications after controlling for diabetes duration, glucose, urea, eGFR, ESR, leukocyte count, and UACR (adjusted OR = 1.956; 95% CI: 1.479–2.587; *p* < 0.001). The results of Model 2 are presented in Table 5.

**Figure 3:**
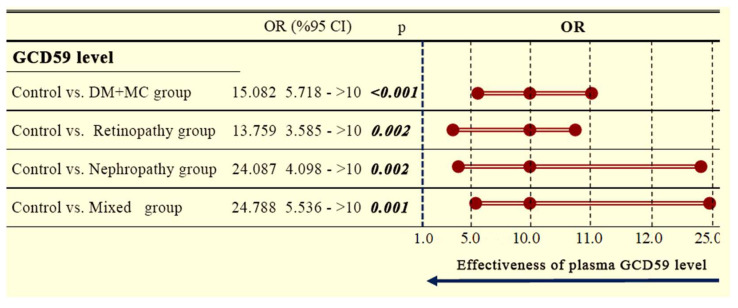
Forest plot showing the odds ratios and 95% confidence intervals for plasma GCD59 levels in diabetic patients with various microvascular complications compared to healthy controls. DM + MC: diabetes mellitus with microvascular complications; OR = odds ratio; CI = confidence interval; GCD59: glycated CD59. Statistically significant *p*-values were shown in bold.

Plasma GCD59 levels were evaluated for their discriminatory capacity between groups using forest plot analysis. Compared to healthy controls, DM + MC patients exhibited significantly higher GCD59 levels. The overall odds ratio (OR) for the DM + MC group was 15.082 (95% confidence interval [CI]: 5.718–∞; *p* < 0.001). Subgroup analyses showed similarly elevated ORs in patients with retinopathy (OR: 13.759, 95% CI: 3.585–∞; *p* = 0.002), nephropathy (OR: 24.087, 95% CI: 4.098–∞; *p* = 0.002), and mixed complications (OR: 24.788, 95% CI: 5.536–∞; *p* = 0.001), as illustrated in the forest plot (Figure 3).

**Figure 4:**
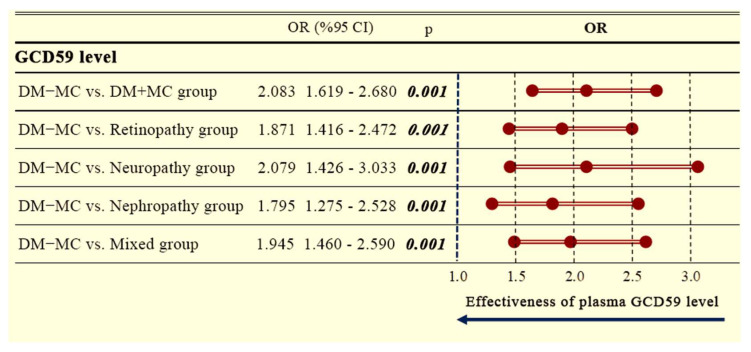
Forest plot displaying the comparative odds ratios and 95% confidence intervals between diabetic patients with and without microvascular complications, stratified by complication subtype. DM − MC: diabetes mellitus without microvascular complications; DM + MC: diabetes mellitus with microvascular complications; OR = odds ratio; CI = confidence interval; GCD59: glycated CD59. Statistically significant *p*-values were shown in bold.

Additionally, a separate forest plot analysis comparing DM − MC patients to DM + MC patients revealed significantly increased GCD59 levels across all complication subgroups. The overall OR for the DM + MC group was 2.083 (95% CI: 1.619–2.680; *p* = 0.001). Elevated ORs were also observed for retinopathy (OR: 1.871, 95% CI: 1.416–2.472; *p* = 0.001), neuropathy (OR: 2.079, 95% CI: 1.426–3.033; *p* = 0.001), nephropathy (OR: 1.795, 95% CI: 1.275–2.528; *p* = 0.001), and mixed complications (OR: 1.945, 95% CI: 1.460–2.590; *p* = 0.001), as shown in Figure 4.

Spearman correlation analysis was performed to evaluate the relationship between plasma GCD59 levels and systemic inflammatory markers. Statistically significant positive correlations were observed between GCD59 and leukocyte count (r = 0.375, *p* < 0.001) and CRP (r = 0.118, *p* = 0.045). A borderline correlation was also identified with ESR (r = 0.114, *p* = 0.055). These data are presented in Table 6.

## 4. Discussion

This study provides compelling evidence that plasma GCD59 is strongly associated with the presence of microvascular complications in patients with T2DM. Importantly, GCD59 levels were significantly higher in diabetic patients with microvascular complications (DM + MC) compared to both diabetic patients without complications (DM − MC) and healthy controls, even though classical glycemic markers such as HbA1c and fasting glucose showed no significant differences. These findings suggest that GCD59 reflects pathological processes beyond hyperglycemia alone, potentially capturing key mechanisms that include endothelial dysfunction, chronic inflammation, and complement system dysregulation, which are not adequately detected by conventional metabolic indices.

This finding holds particular significance, as many patients with well-controlled HbA1c levels still progress to develop microvascular complications. This underscores the urgent need for biomarkers that capture tissue-level injury rather than reflecting only systemic glycemic control. Our findings are consistent with previous studies, indicating that GCD59, a glycated and inactivated form of the complement regulatory protein CD59, plays a pivotal role in driving complement overactivation, the formation of the MAC, and subsequent microvascular injury within diabetic tissues [24].

Importantly, ROC curve analysis confirmed the discriminative capacity of serum GCD59. A cut-off value of 2.210 ng/mL yielded an AUC of 0.946 for differentiating DM + MC patients from healthy controls, with high sensitivity and specificity (89.0 percent and 97.6 percent, respectively). Additionally, a higher cut-off of 3.410 ng/mL differentiated patients with and without complications (DM − MC versus DM + MC), achieving an AUC of 0.849 with solid diagnostic performance. These findings align with previous research, particularly the study of Qin et al., who proposed that GCD59 plays a role in glycation-associated complement activation, contributing to the vascular complications observed in diabetes [12]. Similarly, Acosta and colleagues further clarified the molecular mechanisms linking CD59 dysfunction to complement-mediated vascular injury in diabetes [11]. Building on these mechanistic insights, Ghosh et al. developed a high-specificity assay for measuring GCD59 in plasma, establishing it as a novel biomarker for detecting abnormal glucose metabolism [25].

In a later study, Ghosh and colleagues evaluated plasma GCD59 levels in pregnant women and demonstrated that it was a sensitive marker for detecting gestational glucose intolerance, even in the absence of overt hyperglycemia, highlighting its role in early metabolic dysregulation and screening utility beyond type 2 diabetes [26]. Similarly, Bogdanet et al. confirmed the clinical applicability of GCD59 in gestational diabetes, noting its responsiveness to dynamic glycemic changes during pregnancy and its potential in follow-up strategies [4]. Furthermore, Mellbin et al., in the DIGAMI 2 trial, linked systemic complement activation, potentially downstream of CD59 inactivation, to poor cardiovascular outcomes in type 2 diabetes patients’ post-myocardial infarction [10]. Together, these prior findings support the notion that GCD59 may serve as a more sensitive and stable biomarker for microvascular dysfunction than conventional glycemic parameters alone, and the high sensitivity and specificity demonstrated in our analysis further underscore its potential as a valuable screening and risk stratification tool.

Notably, our subgroup analyses revealed that elevated GCD59 levels were consistently observed across microvascular complication types, including retinopathy, nephropathy, and neuropathy, with no significant differences among them. This suggests that GCD59 may reflect a shared pathogenic pathway across different vascular beds, likely linked to systemic complement dysregulation rather than organ-specific mechanisms. This interpretation is supported by histopathological and experimental studies demonstrating that hyperglycemia-induced glycation leads to the inactivation of CD59, a key regulator that prevents the formation of the MAC [12]. Inactivation of CD59 facilitates MAC deposition, which contributes to endothelial damage and inflammation. MAC accumulation has been demonstrated in renal glomeruli, retinal vessels, and peripheral nerves in both human and experimental diabetic models [27,28,29,30]. These findings suggest that GCD59 may act as a systemic biomarker for complement-mediated microvascular injury, providing a deeper understanding of its pathophysiological role and further supporting its clinical relevance. Moreover, multivariate logistic regression analyses confirmed that plasma GCD59 remained independently associated with microvascular complications even after adjusting for a comprehensive panel of potential confounders, including diabetes duration, metabolic indices, inflammatory markers, and renal parameters. These findings suggest that GCD59 may serve as a robust predictive marker even when traditional indicators such as HbA1c and UACR are taken into account.

In addition to GCD59, our study identified significant alterations in inflammatory and metabolic parameters. Elevated levels of CRP, ESR, and leukocyte counts were consistently observed in diabetic patients with microvascular complications. These findings are consistent with the research of Pickup et al., who demonstrated that type 2 diabetes is characterized by chronic low-grade inflammation, reflected by elevated acute-phase reactants and interleukin-6, contributing to metabolic dysfunction [31]. Similarly, Forbes and Cooper elaborated on the central role of inflammatory pathways and oxidative stress in driving the pathogenesis of diabetic microvascular complications, reinforcing the relevance of systemic inflammatory markers in disease progression [32]. Furthermore, we demonstrated weak significant positive correlations between plasma GCD59 levels and systemic inflammatory markers, including CRP, ESR, and leukocyte count. These associations provide further support that GCD59 may reflect a pro-inflammatory vascular environment and potentially interact with immune-mediated mechanisms contributing to diabetic microvascular damage.

Our findings demonstrate that elevated plasma GCD59 levels in patients with diabetic microvascular complications position this molecule as both a promising biomarker and a potential therapeutic target. The systemic association between GCD59 and microvascular involvement suggests that complement dysregulation plays a central role in diabetic vasculopathy. Preclinical studies have shown that gene therapy delivering soluble CD59 can reduce MAC deposition and protect retinal vasculature in diabetic models, while CD59-deficient mice display worsened vascular pathology, underscoring the protective effects of complement regulation [33,34]. Importantly, current clinical screening strategies for microvascular complications largely rely on late-stage detection methods, such as fundoscopic examinations, urinary albumin measurements, and nerve conduction studies, which typically identify damage only after irreversible injury has occurred. Incorporating plasma GCD59 measurements into clinical practice could improve early risk assessment by identifying high-risk individuals before clinical signs appear, thereby guiding earlier intervention and management strategies.

In addition to its role in microvascular complications, the potential utility of GCD59 as a predictive marker for macrovascular events such as cardiovascular disease remains to be clarified. Prior research has indicated that complement activation and endothelial dysfunction mechanisms in which GCD59 may play a role are also central to the pathogenesis of atherosclerosis and cardiovascular risk in diabetes [34]. Moreover, it is plausible that GCD59 levels may vary with the progression of microvascular disease severity, such as advancing stages of diabetic retinopathy, although longitudinal validation is lacking. Emerging evidence also supports the notion that glycation-related biomarkers may reflect responsiveness to glycemic control strategies [9,35]. These aspects merit dedicated investigation in future multicenter, longitudinal studies incorporating serial GCD59 measurements and outcome tracking.

Although GCD59 shows strong promise as a biomarker for microvascular complications in diabetes, several important issues must be addressed before it can be widely used in clinical practice. Currently, GCD59 is measured using research-grade ELISA kits that lack international standardization. Variations in antibody specificity, calibration methods, and laboratory conditions can result in inconsistent results between centers, limiting comparability across studies [13,25]. In addition, there is no universally accepted diagnostic threshold. Reported cut-off values differ by population and clinical context; for instance, 2.2 ng/mL was proposed for gestational diabetes screening, whereas our study identified 3.4 ng/mL as optimal for detecting microvascular complications in T2DM (26). Large-scale, multiethnic cohort studies are needed to validate these thresholds and to define age- and sex-specific reference ranges. From a cost-effectiveness perspective, although ELISA is relatively affordable compared to other proteomic platforms, implementing GCD59 in routine settings will require automation and integration into high-throughput systems. Recent studies in gestational diabetes have demonstrated that such platforms are feasible and effective for clinical use [4]. Ultimately, ongoing efforts in assay standardization and prospective validation will be crucial for transitioning GCD59 from research to routine clinical diagnostics.

This study has several limitations. First, it was conducted at a single center with a relatively homogeneous patient population, which may restrict external validity. Second, the cross-sectional design precludes causal inference between elevated GCD59 and microvascular complications. Third, although multivariate models adjusted for diabetes duration and major clinical covariates, residual confounding arising from self-reported medication adherence, treatment heterogeneity, or unmeasured lifestyle factors (e.g., diet, physical activity) cannot be excluded. Fourth, the modest sample size may have limited statistical power to detect subtle subgroup effects. Fifth, Net Reclassification Improvement (NRI) analysis, which could more precisely quantify the incremental value of GCD59, was not feasible because predefined risk categories and longitudinal outcome data were unavailable. Sixth, complement activation products such as the MAC were not measured, limiting mechanistic insight. Finally, the absence of longitudinal follow-up prevents determination of whether elevated GCD59 precedes the development of microvascular complications or merely reflects established disease. Large, multiethnic, prospective cohorts with serial GCD59 measurements, predefined risk strata, NRI analysis, and mechanistic biomarkers are required to validate and extend these findings.

In conclusion, this study identifies GCD59 as a promising biomarker associated with microvascular complications in diabetes, underscoring its potential to enhance clinical risk assessment and guide earlier interventions. By providing additional prognostic value beyond traditional markers, GCD59 measurement could help refine patient management strategies and ultimately contribute to reducing the burden of diabetic microvascular disease. Future longitudinal and multicenter investigations are needed to validate these findings, clarify the temporal relationship between GCD59 elevation and the development of microvascular complications, and determine whether therapeutic targeting of glycation or complement pathways can meaningfully improve clinical outcomes.

## Figures and Tables

**Table 1:** Demographic, anthropometric, and clinical data of the patients.

Parameters	Healthy Control Group 1 (*n*: 82)	DM − MC Group 2 (*n*: 82)	DM + MC Group 3 (*n*: 82)	*p*
Age, years	59 (48–63)	59.5 (50–66)	60.5 (53–68)	0.098
Gender, *n* (%)				
- Female	49 (59.8)	39 (47.6)	41 (50.0)	0.254
- Male	33 (40.2)	43 (52.4)	41 (50.0)	
Weight, kg	74 ^¥§^ (63–81)	78 (70–85)	81.5 (72–91)	<0.001
BMI, kg/m^2^	26.8 ^¥§^ (24–28)	28.6 (25–32)	29.4 (26–34)	<0.001
DM duration, years	-	7 (5–14)	15 (10–20)	<0.001
SBP, (mmHg)	120 ^¥§^ (114–124)	130 (120–132)	130 (120–140)	<0.001
DBP, (mmHg)	74 ^¥§^ (67–78)	83 (75–90)	80 (70–90)	<0.001
Comorbidity, *n* (%)				
- Hypertension	-	48 (58.5)	55 (67.1)	0.258
- Hyperlipidemia	-	41 (50.0)	42 (51.2)	0.876
Ischemic heart disease	-	11 (12.8)	16 (19.5)	0.077
Smoking status, *n* (%)				
- Never	82 (100)	62 * (75.6)	71 * (86.6)	<0.001
- Current smoker	-	11 (13.4)	8 (9.8)	
- Ex-smoker	-	9 (11.0)	3 (3.7)	
- Smoking exposure (pack-years)	-	25 (15–36)	40 (22–58)	0.172
Medication, *n* (%)	-			
- Metformin	-	43 (79.6)	57 (78)	0.959
- DPP4 inhibitors	-	27 (50)	39 (53)	0.695
- SGLT2i	-	24 (44)	39 (53)	0.300
- Antihypertensives	-	12 (22.2)	26 (35.6)	0.072
- Lipid-lowering drugs	-	14 (25.9)	30 (41)	0.053

DM − MC: diabetes mellitus without microvascular complications; DM + MC: diabetes mellitus with microvascular complications; BMI: body mass index; SBP: systolic blood pressure; DBP: diastolic blood pressure; DPP4: dipeptidyl peptidase 4; SGLT2İ: sodium-glucose cotransporter-2 inhibitors. Data were presented as median (interquartile range). Categorical parameters were expressed as *n* (percentage). Post hoc analysis was performed with Dunn’s method. * Difference from the control group, *p* < 0.05; ^¥^ difference from the DM − MC group, *p* < 0.05; ^§^ difference from the DM + MC group, *p* < 0.05.

**Table 2:** A comparison of plasma GCD59 levels and relevant laboratory parameters between groups.

Parameters	Healthy Control Group 1 (*n*: 82)	DM − MC Group 2 (*n*: 82)	DM + MC Group 3 (*n*: 82)	*p*
GCD59, ng/mL	1.2 ^§^ (1–1.4)	1.9 *^§^ (1–2.9)	4.5 (3.2–7.1)	<0.001
FBG, mg/dL	89 ^¥§^ (83–96)	143 ^§^ (113–200)	196 (126–248)	<0.001
HOMA-IR	1.7 ^¥§^ (1.2–2.1)	4.3 (2.4–7.9)	5.2 (2.1–8.2)	<0.001
HbA1c, %	5.1 ^¥§^ (4.8–5.5)	7.8 (6.8–8.9)	8.3 (7.3–9.5)	<0.001
Creatinine, mg/dL	0.73 ^¥§^ (0.6–0.9)	0.80 (0.7–0.9)	0.84 (0.7–1.1)	0.002
eGFR, mL/min/1.73 m^2^	99.7 (90–107)	94.6 * (84–103)	87.5 *^¥^ (63–99)	<0.001
ALT, U/L	17 (12–21)	17 (13–24)	17 (14–21)	0.546
TC, mg/dL	195 (169–230)	185 (161–222)	183 (163–211)	0.111
Triglyceride, mg/dL	141.5 (106–186)	147.5 (115–224)	156 (100–222)	0.162
HDLc, mg/dL	46 (40–55)	42.5 (38–51)	45 (38–54)	0.189
LDLc, mg/dL	124 (91–137)	112 * (80–133)	102 * (80–133)	0.001
ESR, mm/h	6 ^¥§^ (3–9)	7.3 ^§^ (4–11)	11 (5–17)	<0.001
CRP, mg/L	1.5 ^¥§^ (0.9–2.4)	2.5 (1–6)	2.4 (1.4–8)	<0.001
Leukocyte, 10^3^/UL	7.2 ^§^ (6–8.7)	7.6 ^§^ (6.6–9.2)	8.9 (7.7–10.5)	<0.001
Hemoglobin, g/dL	13.7 (13.1–14.6)	14.1 (13.2–14.9)	13.9 (12.7–15)	0.319
Platelet, 10^3^/uL	263 (230–307)	270.5 (221–321)	272 (220–330)	0.959
TSH, mIU/L	1.9 (1–2.9)	1.7 (1.1–2.5)	1.9 (1.4–2.8)	0.387
UACR, mg/g	2.7 ^§^	9.9 *^§^	27	<0.001

GCD59: glycated CD59; FBG: fasting blood glucose; HbA1c: hemoglobin A1c; HOMA-IR: homeostasis model assessment of insulin resistance; LDLc: low-density lipoprotein cholesterol; HDLc: high-density lipoprotein cholesterol; TSH: thyroid-stimulating hormone; eGFR: estimated glomerular filtration rate; ALT: alanine aminotransferase; TC: total cholesterol; CRP: C-reactive protein; ESR: erythrocyte sedimentation rate. Post hoc analysis was performed with Dunn’s method. UACR: urine albumin-to-creatinine ratio. * Difference from the control group, *p* < 0.05; ^¥^ difference from the DM − MC group, *p* < 0.05; ^§^ difference from the DM + MC group, *p* < 0.05.

**Table 3:** A comparison of plasma GCD 59 levels across study groups and microvascular complication subtypes.

	Plasma GCD59 Level	*p*
Healthy Control ^23456^	1.2	<0.001
DM − MC ^3456^	1.9	<0.001
Retinopathy	4.6	<0.001
Neuropathy	4.3	<0.001
Nephropathy	4.3	<0.001
Mixed	4.5	<0.001

Post hoc analysis was performed with Dunn’s method. ^2^ Difference from the DM − MC group, *p* < 0.05; ^3^ difference from the retinopathy group, *p* < 0.05; ^4^ difference from the neuropathy group, *p* < 0.05; ^5^ difference from the nephropathy group, *p* < 0.05; ^6^ difference from the mixed group, *p* < 0.05; DM − MC: diabetes mellitus without microvascular complications; GCD59: glycated CD59; mixed group = patients with ≥2 concurrent microvascular complications.

**Table 4:** Multivariate logistic regression analysis of microvascular complications in Model 1 (Control vs. DM + MC).

Variables	Univariate Model				Multivariate Model			
	OR	95% CI		*p*	OR	95% CI		*p*
Age, years	1.033	1.002	1.064	0.038				
BMI, kg/m^2^	1.257	1.144	1.381	<0.001				
Systolic BP, mmHg	1.158	1.101	1.217	<0.001				
Diastolic BP, mmHg	1.100	1.057	1.146	<0.001				
Glucose, mg/dL	1.100	1.057	1.143	<0.001	1.136	1.042	1.238	0.004
HbA1c, %	>100	11.75	>100	<0.001				
eGFR, mL/min/1.73 m^2^	0.968	0.951	0.985	<0.001				
LDL, mg/dL	0.983	0.973	0.992	<0.001				
ESR, mm/h	1.123	1.064	1.185	<0.001				
CRP, mg/L	1.128	1.037	1.228	0.005				
WBC, 10^3^/μL	1.698	1.377	2.095	<0.001				
UACR, mg/g	1.155	1.085	1.230	<0.001				
GCD59, ng/mL	15.09	5.719	39.79	<0.001	21.48	3.505	131.62	0.001

BMI: body mass index; BP: blood pressure; eGFR: estimated glomerular filtration rate; LDL: low density lipoprotein; ESR: erythrocyte sedimentation rate; CRP: C-reactive protein; WBC: white blood cell count; UACR: urinary albumin-to-creatinine ratio; GCD59: glycated CD59; OR: odds ratio; CI: confidence interval. Multivariable logistic regression was performed using a stepwise forward likelihood ratio method after assessment of collinearity among candidate variables.

**Table 5:** Multivariate logistic regression analysis of microvascular complications in Model 2 (DM − MC vs. DM + MC).

Variables	Univariate Model				Multivariate Model			
	OR	95% CI		*p*	OR	95% CI		*p*
DM duration, years	1.106	1.057	1.158	<0.001	1.086	1.022	1.154	0.008
Glucose, mg/dL	1.005	1.001	1.009	0.013				
Urea, mg/dL	1.045	1.018	1.073	<0.001				
eGFR, mL/min/1.73 m^2^	0.976	0.960	0.992	0.003				
ESR, mm/h	1.038	1.002	1.075	0.036	1.060	1.011	1.112	0.016
WBC, 10^3^/μL	1.298	1.106	1.524	0.001				
UACR, mg/g	1.056	1.029	1.083	<0.001				
GCD59, ng/mL	2.083	1.619	2.680	<0.001	1.956	1.479	2.587	<0.001

eGFR: estimated glomerular filtration rate; ESR: erythrocyte sedimentation rate; WBC: white blood cell count; UACR: urinary albumin-to-creatinine ratio; GCD59: glycated CD59: OR: odds ratio; CI: confidence interval. Multivariable logistic regression was performed using a stepwise forward likelihood ratio method after assessment of collinearity among candidate variables.

**Table 6:** The correlation between plasma GCD59 levels and inflammatory markers.

Parameters	r	*p*
CRP, mg/L	0.118	0.045
ESR, mm/h	0.114	0.055
Leukocyte, 10^3^/µL	0.375	<0.001

CRP, C-reactive protein; ESR, erythrocyte sedimentation rate. Correlation analysis was performed using Spearman’s rank correlation coefficient.

## Data Availability

The data that support the findings of this study are available from the corresponding author upon reasonable request.

## Abbreviations

T2DM	Type 2 diabetes mellitus
GCD59	Glycated CD59
DM − MC	Diabetes mellitus without microvascular complications
DM + MC	Diabetes mellitus with microvascular complications
BMI	Body mass index
HbA1c	Glycated hemoglobin
HOMA-IR	Homeostasis model assessment of insulin resistance
CRP	C-reactive protein
ESR	Erythrocyte sedimentation rate
UACR	Urine albumin-to-creatinine ratio
ELISA	Enzyme-linked immunosorbent assay
MAC	Membrane attack complex
MNSI	Michigan neuropathy screening index
TCNS	Toronto clinical neuropathy score
CKD-EPI	Chronic kidney disease epidemiology collaboration
ROC	Receiver operating characteristic
AUC	Area under the curve
PPV	Positive predictive value
NPV	Negative predictive value
CI	Confidence interval
